# Liquid chromatography-tandem mass spectrometry (LC-MS/MS) determination of cantharidin in biological specimens and application to postmortem interval estimation in cantharidin poisoning

**DOI:** 10.1038/s41598-020-67278-x

**Published:** 2020-06-26

**Authors:** Youyou Zhang, Liang Liu, Liang Ren

**Affiliations:** Department of Forensic Medicine, Tongji Medical College, Huazhong University of Science and Technology, Wuhan, 430030 China

**Keywords:** Biomarkers, Medical research

## Abstract

A rapid, sensitive liquid chromatography-tandem mass spectrometry (LC-MS/MS) method was developed and validated for the determination and quantification of cantharidin in rats liver and kidney. After grinding with methanol, the supernatant was determined by LC-MS/MS using an Thermo Accucore C18 column (100 mm×2.1 mm, 2.6 μm) with a gradient elution of 0.1% formic acid and 0.1% acetonitrile, and in the subsequent analysis using selected reaction monitoring mode, three ion transitions were monitored for analyte. The limit of detection (LOD) was 0.741 ng/ml and the limit of quantitation (LOQ) was 2.471 ng/ml. Good linearity (R^2^ = 0.9998) was observed for the analyte over the linear range (5–400 ng/ml). The LC-MS/MS method was applied to the analysis of rats liver and kidney in different postmortem intervals (6 h, 12 h, 24 h, 48 h, 72 h and 168 h after death) after a single dose (4 mg/kg) of cantharidin administration by gavage. At 72 h after death, the cantharidin concentration in livers and kidneys were significantly higher than that in other postmortem intervals. Linear regression equations between postmortem interval and lg postmortem cantharidin concentration in rats liver and kidney were Y = 0.007455*X + 1.332(R^2^ = 0.863) and Y = 0.002689*X + 1.433 (R^2^ = 0.115) respectively. The animal experiment demonstrated LC-MS/MS method can be used to determine the postmortem cantharidin concentration in rats liver and kidney and the determination of cantharidin in the rats liver after death has potential value for postmortem interval estimation in cantharidin poisoning.

## Introduction

Cantharidin, as the principal active ingredient of traditional Chinese medicine mylabris, was firstly discovered and used as anticancer drug in China^1^. What is more, cantharidin-based pharmaceutical preparations, such as *Fufangbanmao* caspsules and *Aidi* injection, are now widely used in clinical treatment for cancers with good therapeutic efficacy^2–4^. However, previous studies on the clinical efficacy and safety of cantharidin indicated that cantharidin had risk of hematotoxicity, gastrointestinal toxicity, liver or renal injury, neurotoxicity, cardiotoxicity and so on^5–7^. Furthermore, many village doctors use the unprocessed mylabris for treatment in patients and the effects of different processing to pharmacodynamic action of canthardin were different^8,9^. All of these lead to the lack of unified evaluation criteria for clinical efficacy and safety. As we know, the toxic dosage of cantharidin was similar to therapeutic dosage^10^ and the potential risk of cantharidin poisoning far outweighs any potential benefit of therapeutic efficacy was caused by the unreasonable use or inappropriate medication in patients. Evenmore, cantharidin poisoning can lead to muti-organ failure even death^8^ and the following conditions of cantharidin poisoning were also found in forensic identification center^8,11–14^ such as patients who died after ingestion of cantharidin as aphrodisiac, ingested blister beetles accidentally, suicide by ingesting mylabris, homicide by poisoning, and so on. Consequently, the detection and evaluation of postmortem cantharidin concentration is of great practical significance, which play a critical role in the forensic identification work in cantharidin poisoning.

Toxicological analysis is important to the forensic diagnosis on death after cantharidin poisoning especially in the deaths of suicide or homicide, which can provide clue to the analysis of poisoning cases and would be helpful for the postmortem interval (PMI) estimation. The chemical structure of cantharidin was 2,3-dimethyl-7-oxabicyclo^1,2^heptane-2,3-dicarboxylic anhydride, which release from the blister beetles and was isolated by Robiquet^15^. In previous studies, gas chromatography (GC)^16,17^, gas chromatography-mass spectrometry(GC-MS)^18–20^, high-performance liquid chromatography(HPLC)^21,22^ and liquid chromatography tandem mass spectrometry(LC-MS)^23^ have been the methods used for cantharidin determination in plasma and pharmacokinetic study in animal models. All of the methods proved to have high precision, accuracy and sensitivity, and were suitable for cantharidin determination in plasma. What is important, LC-MS/MS method with the characteristics of rapid, sensitive and specific, which offer an added advantage for toxin determination in diminutive levels has been proved by some previous studies^24,25^. As we know, sample preparation plays an important role in the process of chemical analytical. In recent years, the extraction and separation technologies including membrane separation technology^26^, supercritical fluid extraction^27^, and electrospray ionization-mass spectrometer system^28^ have been developed to increase the extraction efficiency or enhance the analyte signal strength. These methods are expected to be applied to the forensic identification work for forensic toxicological analysis and to the drug concentration analysis in clinic practice. Cantharidin, as one of the traditional anti-cancer/skin-disease medicine, the pharmacokinetic characteristics especially the postmortem changes of it is still unknown. Previous studies were mainly focused on the determination of plasma cantharidin after administration, and there was no literature reported to measure cantharidin in the biological samples after administration. Consequently, the study on the postmortem cantharidin determination in biological samples will enrich the pharmacokinetic characteristics of cantharidin and provide the pharmacokinetic parameters for the rational use and postmortem redistribution of cantharidin.

On the basis of the analyses above, we aim to utilize the analytical method of LC-MS to study the postmortem concentrations of cantharidin after administration in rats liver and kidney in this study. And our aim was also to evaluate the postmortem concentration of cantharidin in different PMI periods taken from the biological samples and to compare the difference between the samples of liver and kidney. All of these will provide a better understanding of the postmortem distribution of cantharidin and the relationship between postmortem cantharidin concentration and PMI estimation.

## Materials and methods

### Chemical and reagents

Cantharidin (purity ≥98%), methanol (MeOH), acetonitrile (ACN), and formic acid were purchased from Shanghai Aladdin Bio-Chem Technology Co., Ltd. (Shanghai, China). Ultrapure water (high- performance liquid chromatography [HPLC] grade) was supplied by the Direct-Q5 water purification system (Merck Millipore, Germany).

### Animals

Eighteen specific pathogen-free (SPF) male Sprague-Dawley (SD) rats (weighing 200–230 grams) were purchased from the Vitalriver Experimental Animal Technology Co. Ltd. (Beijing, China) and were maintained on a 12-hour light/dark cycle in a controlled temperature (20–22°C) and humidity (40–50%) environment, which had unlimited access to food and water. Rats were acclimatized to the animal facility for a week and then were administrated cantharidin (4 mg/kg) by method of intragastric, all rats died spontaneously within 6 h and were stored in an environment with constant temperature and humidity (20 °C temperature, relative humidity of 50%). The rats were randomly divided into six groups based on the time of liver and kidney collection (6 h, 12 h, 24 h, 48 h, 72 h and 168 h after death; n = 3 per group). The samples of liver and kidney were frozen at −80 °C until analysis. All animal protocols were performed in accordance with the guide for the care and use of laboratory animals published by the US National Institutes of Health (NIH Publication No. 85–23, revised 1996) and were approved by the Huazhong university of science and technology animal welfare committee.

### Standard solution, quality control of samples and sample preparation

Cantharidin standard solution was dissolved in MeOH to obtain a 2 mg/ml stock solution, the stock solution was stored at −20 °C and was freshly prepared. Then the stock solutions of cantharidin was diluted with MeOH. The concentrations of analytical standard solutions of cantharidin were 400, 200, 80, 40, 10, 5, and 2 ng/ml. And the accuracy of developed analytical method can be significantly enhanced by the calibration curves. Limit of detection (LOD) and limit of quantitation (LOQ) were determined to estimate the sensitivity of the analytical method and calculated as the concentration of the inject sample to yield a signal-to-noise ratio of three and ten, respectively.

Two grams liver/kidney tissue was weighed and homogenized then mixed with 1 ml MeOH for 4 min, then the sample was vortexed for 10 min and centrifuged at 15000 rpm for 10 min. At last, 5 μl of the the aliquot was injected into LC-MS/MS.

### LC-MS analysis

An Ultimate 3000 system equipped with a pump (LPG-3400RS), an autosampler (WPS-3000RS), a column oven (TCC-3000RC), and a VWD-3400RS UV/Vis detector (Thermo Scientific, USA) was used for the chromatographic separation. Data were collected and processed by Xcilabur 3.0 (Thermo Scientific, USA). Separation was carried out on a Thermo Accucore C18 (100 mm × 2.1 mm, 2.6 μm) (Thermo Scientific, USA) at 40 °C, with an injection volume of 5 μl. The mobile phases consisted of 0.1% formic acid (A) and 0.1% acetonitrile (B). The flow rate was 0.4 ml/min, and the gradient program was as follows: 5–95% B (0–2.5 min), 95% B (2.5–3.8 min), 95–5% B (3.8–4 min), and 5% B (4–5 min).

Ionization was achieved using electrospray ionization (ESI) in positive mode with selected reaction monitoring (SRM). Mass spectrometry was performed with an ESI source in the positive-ionization mode with a sheath gas of 50 Arb and aux gas of 15 Arb. The ion spray voltage was set at 4000 V and the source temperature was 350 °C. The parameters for the quantification selected reaction monitoring (SRM) transitions are presented in Table 1.

**Table 1:** The parameters for the quantification selected reaction monitoring transitions.

Analytes	Polarity	Parent (m/z)	Product (m/z)	Collision energy (eV)	Tube lens (V)
			95.207	17	89
Cantharidin	+	197.085	123.139	14	89
			134.996	14	89

### Data analysis

Results are shown as mean ± SEM. Multiple comparisons between groups were analyzed by ANOVA, calibration curve was plotted using a weighted (1/x) least square regression and the linearity of the calibration curve was determined by the correlation coefficients (R^2^). The normal distribution test was conducted in the variables, and the abnormal distribution test was applied to analyze the data after logarithm transition. Then the correlations between the concentration of cantharidin and PMI were evaluated with Pearson’s correlation test. A value of *P* < 0.05 was considered significant. Statistical analysis was performed using SPSS19.0.

### Ethical Approval

All applicable international, national, and/or institutional guidelines for the care and use of animals were followed.

## Results and discussion

### Optimization of analytical method

**Figure 1:**
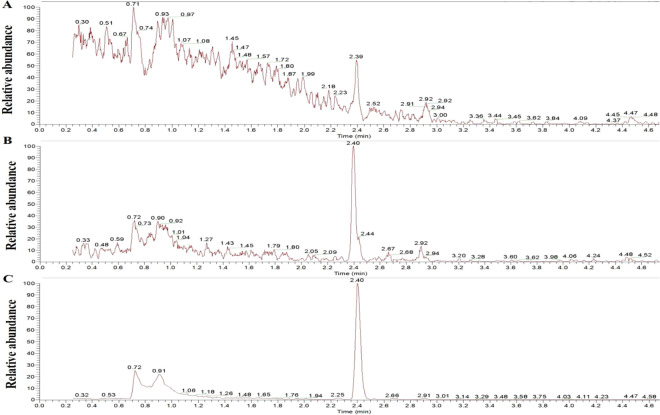
Chromatogram of cantharidin. A, chromatographic separation was carried out on a Welch XB-C18 (2.1 × 50 mm, 1.8μm) at 30 °C with an injection volume of 5 μl; 10 mM ammonium formate containing 0.1% formic acid was used as mobile phase A, 0.1% formic acid and acetonitrile was used as moblie phase B; B, chromatographic separation was carried out on a Thermo Accucore C18 (100 mm × 2.1 mm, 2.6 μm) at 40 °C with an injection volume of 5 μl; 10 mM ammonium formate containing 0.1% formic acid was used as mobile phase A, 0.1% acetonitrile was used as moblie phase B; C, chromatographic separation was carried out on a Thermo Accucore C18 (100 mm × 2.1 mm, 2.6 μm) at 40 °C with an injection volume of 5 μl; 0.1% formic acid (A) was used as mobile phase A and 0.1% acetonitrile was used as moblie phase B.

**Figure 2:**
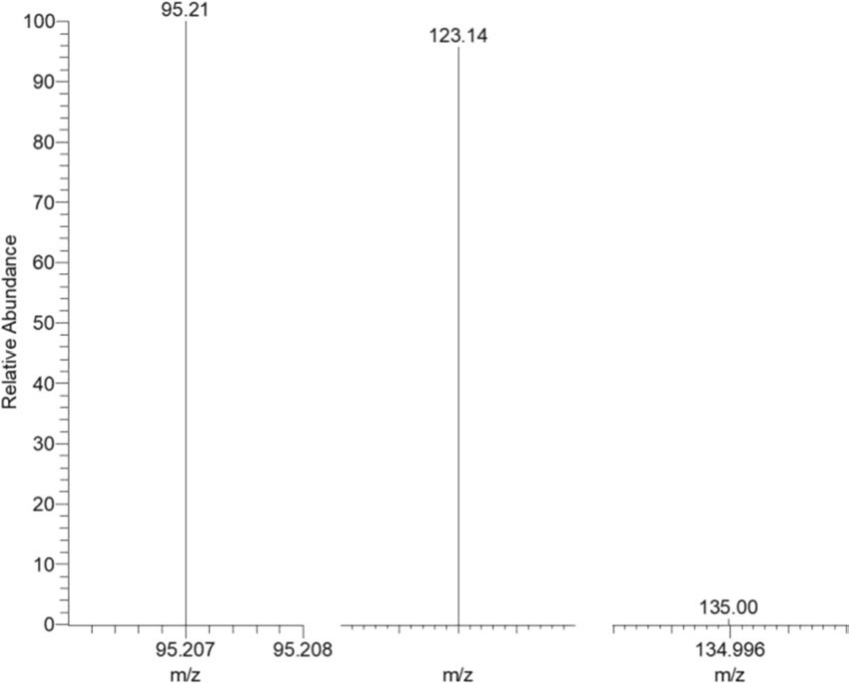
The full-scan production spectrums of cantharidin by injecting the standard solutions into the mass spectrometer.

In our present study, the base peaks of CA (m/z 95.207, 123.139, 134.996) were selected for the quantification of peak area and retention time was 2.4 min under set LC-MS conditions. Firstly, the concentration of analytical standard solutions of cantharidin was 400 ng/ml, and chromatographic separation was carried out on a Welch XB-C18 (2.1 × 50 mm, 1.8μm) at 30 °C with an injection volume of 5 μl. 10 mM ammonium formate containing 0.1% formic acid was used as mobile phase A, 0.1% formic acid and acetonitrile was used as mobile phase B. Figure 1-A showed that the chromatographic of cantharidin by this method and interference peaks were found. Then we replace the chromatographic column with Thermo Accucore C18 (100 mm×2.1 mm, 2.6 μm) at 30 °C with an injection volume of 5 μl, and the chromatographic presenting a few peaks in Fig. 1-B. In order to avoid interference of sample blank, 0.1% formic acid was used as mobile phase A under the optimal chromatographic conditions. The cantharidin had a better response in positive mode under the optimal detection parameters and no obvious interference peaks were observed in Fig. 1-C. The full-scan production spectrums of cantharidin by injecting the standard solutions into the mass spectrometer are shown as Fig. 2. So that the chromatographic column with Thermo Accucore C18 (100 mm×2.1 mm, 2.6 μm) was used for further study and the mobile phases comprised 0.1% formic acid (A) and 0.1% acetonitrile (B), the other parameters were adopted for the recommended value of the instrument.

### Limit of detection, limit of quantitation and linearity

**Figure 3:**
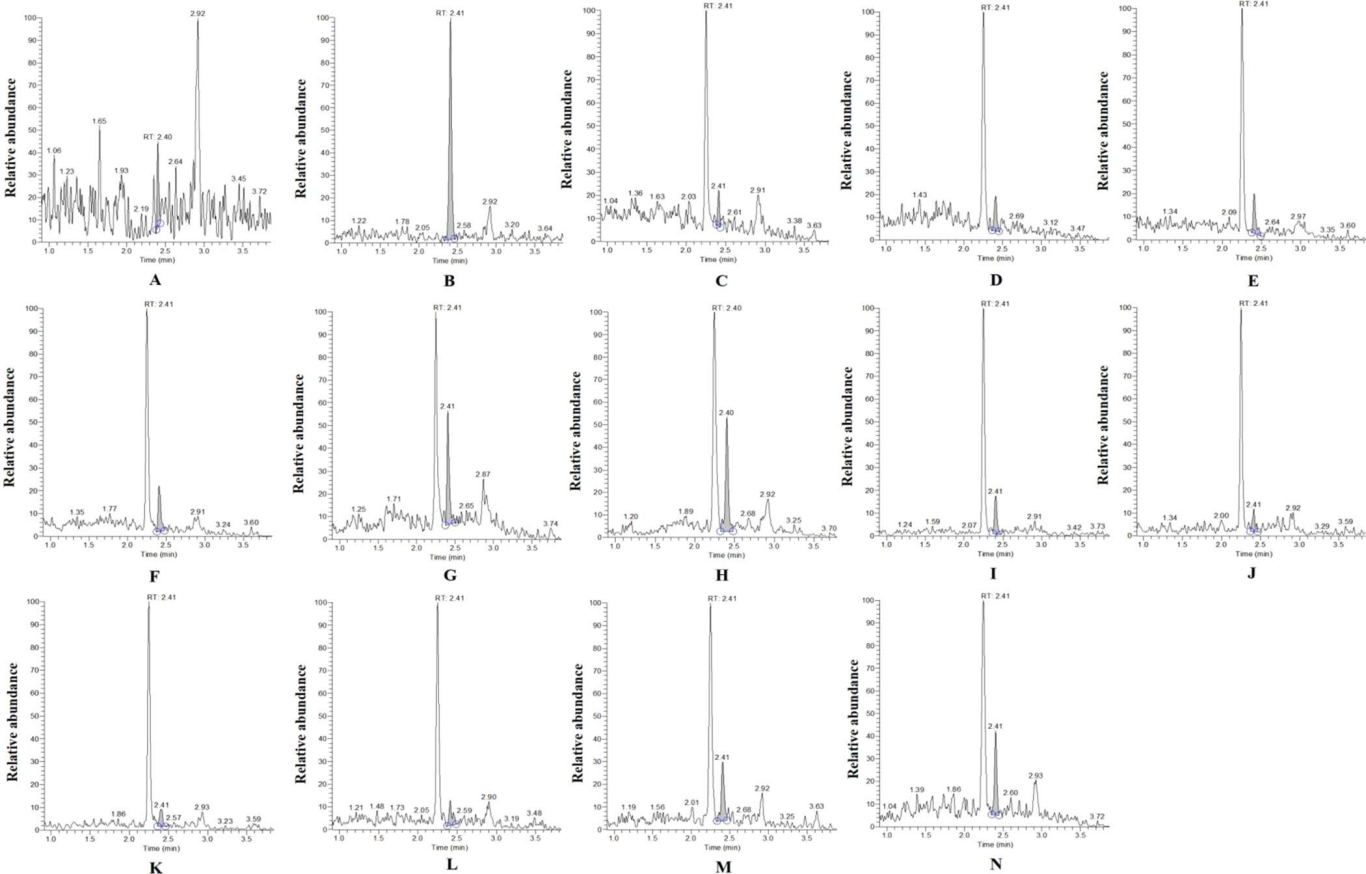
Representative chromatograms obtained from blank control (**A**), blank control spilked with cantharidin (**B**), samples of liver and kidney in different PMI (6 h, 12 h, 24 h, 48 h, 72 h, 168 h) in rats of cantharidin poisoning (**C**-**H**), samples of kidney in different PMI (6 h, 12 h, 24 h, 48 h, 72 h, 168 h) in rats of cantharidin poisoning (**I**-**N**).

SRM scan mode can provide high selectivity in the cantharidin analysis. Representative chromatograms obtained from blank control, blank control spiked with cantharidin, and samples of liver and kidney in different PMI in rats of cantharidin poisoning are shown in Fig. 3. No significant interference or ion suppression was observed at the retention times of cantharidin and ESI.

The LOD and LOQ of the analytical method, calculated as the concentration of the inject sample to yield a signal-to-noise ratio of three and ten, were 0.741 ng/ml and 2.471 ng/ml respectively. And the these results in our study were much lower than previous studies on cantharidin determination in plasma of beagle dogs using GC-MS^19^ and cantharidin determination in human blood using HPLC^22^. Therefore, the proposed method could be used to monitor the cantharidin concentration in biological specimens and the accuracy of the method is satisfactory.

**Figure 4:**
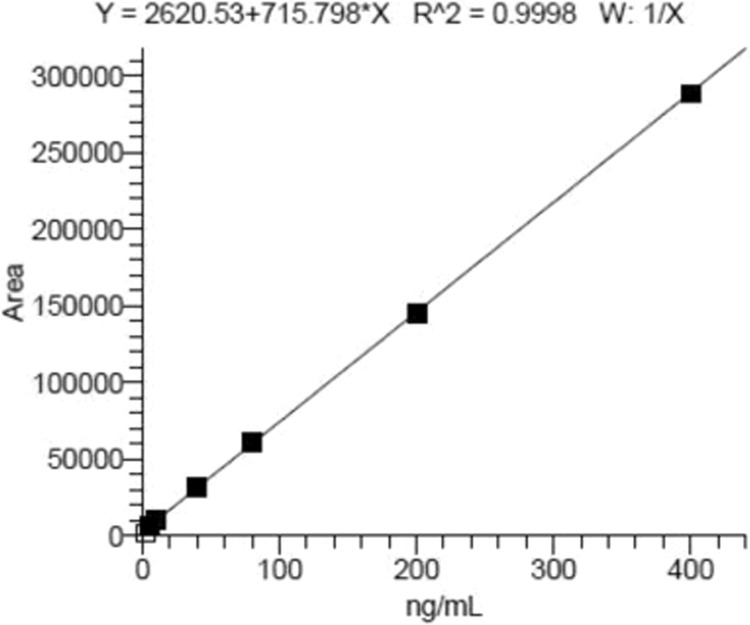
Calibration curve of cantharidin concentrations was established by weighted (w = 1/x) linear regression analysis.

Calibration curve of cantharidin concentration was established by weighted (w = 1/x) linear regression analysis, Fig. 4 showed the linearity (R^2^ = 0.9998) in the range of 5–400 ng/ml and linear regression equation was Y = 2620.53 + 715.798*X where X is the concentration of cantharidin (ng/ml) and Y is the peak area.

### Postmortem distribution of cantharidin in rats liver and kidney

**Figure 5:**
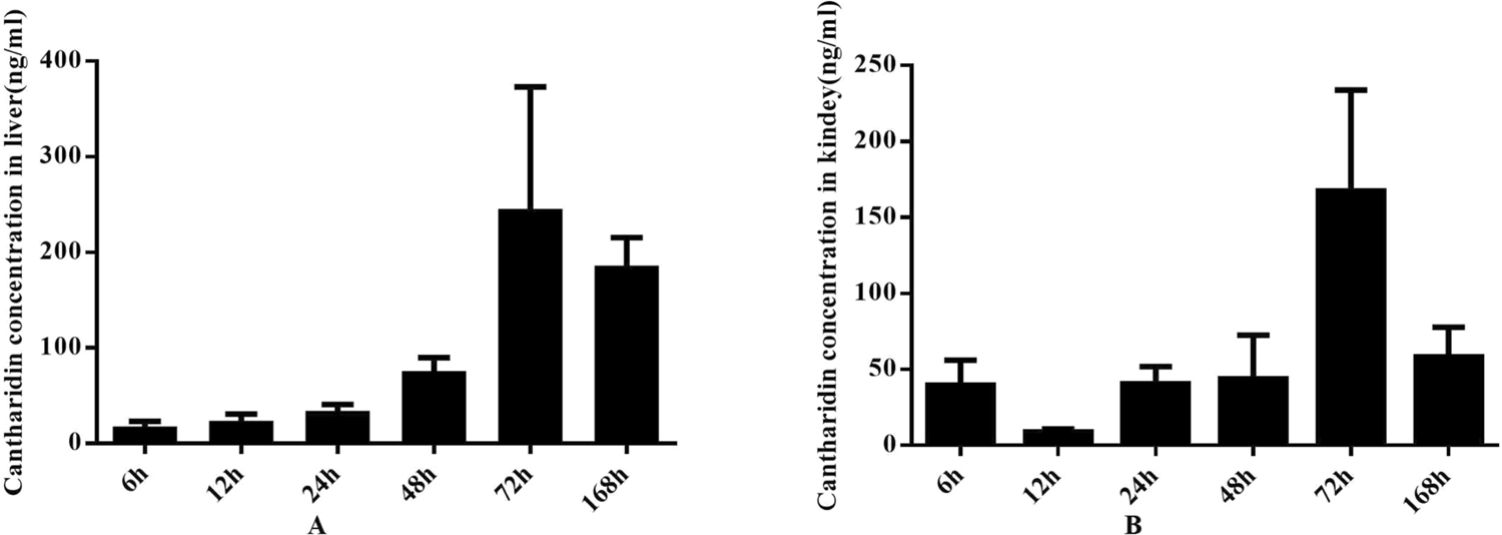
Postmortem distribution of cantharidin in rats liver (**A**) and kidney (**B**).

The analytical method was successfully applied to determine the concentration of cantharidin in rats liver and kidney. Figure 5-A showed that the liver distribution of cantharidin in different PMI periods and the cantharidin concentration in the group of 72 h after death was significantly increased compared with the group of 6 h, 12 h, 24 h, and 48 h. The kidney distribution of cantharidin was shown in Fig. 5-B, the highest cantharidin concentration was the group of 72 h and the statistically significant differences were found between this group and other 5 groups. Factors which influence postmortem redistribution of drugs including diffusion from stomach to nearby organs, cell death, putrefaction, body position and movement after death, drug characteristics^29–31^. In our present study, we analyzed some of influences on postmortem cantaharidin redistribution as follows: Firstly, the anatomical relationships between the liver and the stomach were related to the postmortem cantharidin redistribution, liver is the target of redistribution from the gastrointestinal tract^30^. So we suggest that the passive diffusion from the gastric content into the liver was the main mechanism on postmortem change of cantharidin redistribution in rat liver. Secondly, basic drugs that will be progressively more ionized in an increasingly acidic medium after cell death, which will distribute more readily as a result of being transported in the acidic fluid in which they are dissolved^29^. However, cantharidin is not a basic drug, so we suggest that the cell death may not be involved in the process of postmortem cantharidin redistribution. Thirdly, putrefaction can also contribute to changes in drug concentrations after death^29–31^. Combined that the previous founding that pharmacokinetic profiles of drugs were affected by environment temperature in animal models^32,33^, the rate of postmortem metabolism may also affected by the temperature of corpses preservation^29,30^. In our present study, we found that the highest canthardin concentration in rats liver and kidney was 72 h after death which may be related to the rate of postmortem cantharidin metabolism and body decomposition at the constant temperature of 20 °C and whether there were differences in the postmortem changes of cantharidin concentration among different temperatures of corpses preservation need to be further studied. Fourthly, body position and movement, which may be related to the blood movement after death, have an effect on postmortem redistribution^29,30^. All of the death rats in our study were kept in the same body position and no movement after death, so we suggest that body position and movement was not an important factor that influences the postmortem cantharidin concentration. Finally, canthardin is a lipophilic drug that will concentrate in liver after administration which provides a concentration gradient for passive diffusion after death. Evenmore, Pohland and Bernhard^34^ found that the postmortem redistribution from liver is complex and the process is not as early as redistribution from other organs. The previous findings can explain why the cantharidin concentration decrease in 168 h. Above all, we suggest that the phenomenon that the increase of postmortem cantharidin concentration within 72 h and the decrease in 168 h was affected by the diffusion from stomach to nearby organs, putrefaction, and drug characteristics. These results indicate that it is of great importance to analyze specimens from different postmortem interval in order to detect potential postmortem cantharidin concentration and avoid misinterpretation of results in practical applications.

In this paper, the postmortem cantharidin concentration in rats liver and kidney after cantharidin poisoning could provide much more reliable and valuable data that may help us to understand the postmortem redistribution of cantharidin *in vivo*, which may be helpful to guide the forensic diagnosis in criminal case and medical disputes case in future. In the next research, we will utilize this analytical method to study the other tissues distribution of cantharidin after administration and to reveal the features of postmortem distribution in different tissues.

### Association between postmortem cantharidin concentration and PMI estimation

**Figure 6:**
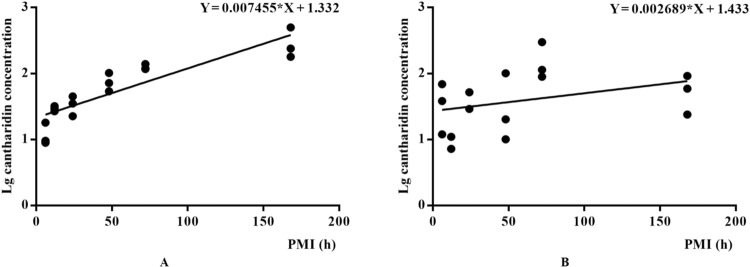
The linear regression equations between PMI and lg postmortem cantharidin concentration in rats liver (**A**) and kidney (**B**).

Based on the postmortem changes of the cantharidin concentration in rats liver and kidney, PMI estimation models were constructed based on the linear regression analysis and the results were shown in Fig. 6. The linear regression equations between PMI and lg postmortem cantharidin concentration in rats liver and kidney were Y = 0.007455*X + 1.332(R^2^ = 0.863) and Y = 0.002689*X + 1.433 (R^2^ = 0.115) respectively. Based on the results of correlation coefficients, we suggest that the determination of cantharidin in the rats liver after death has potential value for PMI estimation after cantharidin poisoning. However, the mechanism of concentration change after death of cantharidin is not clear. Therefore, it is necessary to obtain more comprehensive data based on different cantharidin dosages in our future study.

The homicidal cases of cantharidin poisoning are much inconspicuous, which have brought quite difficulty to crack the criminal case. What is more, toxicological analysis and PMI estimation are important to hunting for clue in these cases. In present study, the postmortem cantharidin determination in liver by LC-MS can be a solution to the problem, the conclusion that obtained in the basis of a small number of samples looks forward to being verified by large sample in further study.

## Conclusions

Here, we present a rapid and sensitive LC-MS/MS method for the cantharidin determination and the method has been successfully demonstrated for postmortem cantharidin determination in rats liver and kidney. Good linearity (R^2^ = 0.9998) was observed for the analyte in the range of 5–400 ng/ml and the LOD and LOQ were 0.741 ng/ml, 2.471 ng/ml respectively. The results of postmortem distribution of cantharidin in rats liver and kidney may help us to understand the postmortem distribution of cantharidin *in vivo* and to guide the forensic diagnosis in criminal cases and in medical disputes cases of death caused by cantharidin poisoning. In addition, PMI estimation models were established based on the postmortem changes of the cantharidin concentration in rats liver and kidney, these results indicate that postmortem cantharidin determination in liver could be a useful tool in PMI estimation. To our best knowledge, this is the first report on the cantharidin determination in biological specimens by LC-MS/MS and application to postmortem interval estimation in cantharidin poisoning, which will provide much more valuable reference to the further work including the subsequent study on postmortem distribution of cantharidin in other tissues and the effects of pharmacokinetic parameters on postmortem redistribution of cantharidin *in vivo*.
